# Quality of Life Following Amputation or Limb Preservation in Patients with Lower Extremity Bone Sarcoma

**DOI:** 10.3389/fonc.2013.00210

**Published:** 2013-08-14

**Authors:** Gary E. Mason, Lele Aung, Sarah Gall, Paul A. Meyers, Robert Butler, Sarah Krüg, Mimi Kim, John H. Healey, Richard Gorlick

**Affiliations:** ^1^Division of Pediatric Hematology/Oncology, Department of Pediatrics, The Children’s Hospital at Montefiore, The Albert Einstein College of Medicine of Yeshiva University, Bronx, NY, USA; ^2^Department of Pediatrics, Memorial Sloan-Kettering Cancer Center, New York, NY, USA; ^3^Parkway Cancer Centre, Gleneagles Hospital, Singapore; ^4^Division of Pediatric Hematology/Oncology, Department of Pediatrics, Oregon Health & Science University, Portland, OR, USA; ^5^Department of Epidemiology and Population Health, The Albert Einstein College of Medicine of Yeshiva University, Bronx, NY, USA; ^6^Department of Surgery, Orthopaedic Surgery Service, Memorial Sloan-Kettering Cancer Center, New York, NY, USA; ^7^Department of Molecular Pharmacology, The Albert Einstein College of Medicine of Yeshiva University, Bronx, NY, USA

**Keywords:** sarcoma, quality of life, amputation, limb salvage, orthopedic outcomes

## Abstract

**Purpose**: Although functional differences have been described between patients with lower extremity bone sarcoma with amputation and limb-preservation surgery, differences have not clearly been shown between the two groups related to quality of life. The purpose of the study was to determine if there is a difference in overall quality of life in lower extremity bone sarcoma survivors related to whether they had an amputation or a limb-preservation procedure while identifying psychological differences for further evaluation. The main hypothesis was that sparing a person’s limb, as opposed to amputating it, would result in a better quality of life.

**Patients and Methods**: Eighty-two long-term survivors of lower extremity bone sarcoma were studied to make a comparison of the overall quality of life, pain assessment, and psychological evaluations in limb preservation and amputation patients. Forty-eight patients with limb preservation and thirty-four patients with amputations were enrolled in the study. Validated psychometric measures including the Quality of Life Questionnaire (QLQ), the Minnesota Multiphasic Personality Inventory, and visual analog scales were utilized.

**Results**: The overall quality of life of patients with limb preservation was significantly higher than patients with amputation (*p*-value < 0.01). Significant differences were noted in the categories of material well-being, job satisfiers, and occupational relations.

**Conclusion**: The overall quality of life of patients with limb-preservation appears to be better than for those patients with amputation based on the QLQ in patients surviving lower extremity bone sarcoma. Further analysis needs to verify the results and focus on the categories that significantly affect the overall quality of life.

## Introduction

Significant improvements have occurred in the treatment of extremity sarcoma patients over the past few decades. The vast majority of bone sarcoma patients underwent amputations for local tumor control a few decades ago. Surgical procedures being utilized for treatment have evolved (1). New surgical techniques such as en bloc resections with replacement by internal prostheses, and multimodality therapy including chemotherapy and limb-preservation surgery have become available (2–7). Limb-preservation surgery includes wide excision of tumor bearing bone and soft tissues and typically implantation of an endoprosthesis. In several trials limb-preservation surgery demonstrated no adverse impact on survival of patients with soft tissue sarcoma and osteosarcoma although the trials were difficult to control (8–10). Currently more than 90% of patients with extremity sarcoma undergo limb-preservation procedures (11). With improved adjuvant therapy, large numbers of patients will be long-term survivors (11–13). It is important to optimize their psychosocial functioning and quality of life which may be affected by surgery. In several prior studies, limb-preservation surgery has not clearly demonstrated a significant difference in the quality of life for patients with bone and soft tissue extremity sarcoma, with the evidence showing mixed results (14–24).

It seems intuitive that limb preservation would preserve better quality of life and psychological health. This difference has yet to be clearly demonstrated in the studies performed and outlined previously. The level of functioning in long-term survivors of sarcoma who undergo either limb preservation or surgery or amputation is generally high. The measures used in the previous studies may not have been sufficiently sensitive to detect a difference or the studies under powered (25, 26). Previous studies of quality of life and psychosocial functioning have demonstrated ongoing changes with increasing time from completion of therapy (26, 27). The purpose of our study was to compare the two groups in a larger patient group with more comprehensive measures. We employed the Minnesota Multiphasic Personality Inventory (MMPI), QOL questionnaires, visual analog pain scales, and a socioeconomic assessment in order to identify more subtle variances in psychological functioning and quality of life and obtain statistical power.

## Patients and Methods

The Division of Orthopaedic Surgery at Memorial Sloan-Kettering Cancer Center (MSKCC) has established and maintains a database of all operative procedures. Over the past 20 years, almost 200 patients have had amputations and over 400 patients have had limb-preservation surgery of the lower extremity for the treatment of bone sarcoma. Approximately 60% of these patients survived their malignancies. Most of these patients are long-term survivors, more than 5 years beyond all planned therapy with no evidence of disease. We offered participation in this study to this group of long-term survivors. The Memorial Hospital Institutional Review Board approved this study. The orthopedic database was reviewed and operative reports were examined to select patients who fit the criteria for eligibility. All subjects were between the ages of 14 and 50 years of age; the ages for which the psychological measure are validated. They were also all native English speaking, due to the fact that the questionnaires administered were only available in English. All patients had a lower extremity bone sarcoma. Forty-two patients who were at least 1 year following limb amputation of the lower extremity and fifty-eight patients who were at least 1 year following limb-preservation surgery of the lower extremity were asked to participate in the study. Eighty-three patients were enrolled in the study. One limb-preservation patient did not complete all the questionnaires so the patient was not included in the analysis. Patients with rotationplasty/turn plasty procedures were excluded as these patients have issues related to both amputation and limb preservation. Patients who underwent limb preservation that failed and had a secondary amputation are not included here. Failures are common and may occur at a rate of 1% per year for distal femoral tumors and higher for tibial tumors (28). Patients were initially contacted by telephone and mail and a signature indicating informed consent was obtained was required for participation. Data regarding age at time of surgery, surgical procedure performed, tumor site, disease, and time elapsed since surgery were obtained by review of the surgery database and operating room reports.

The study involved obtaining quality of life and psychological measure of these patients and comparisons of the measures were used to assess for differences in quality of life and psychological outcome. The psychometric measure required approximately one half day of the subjects time and a majority of the patients were tested in the outpatient facilities of MSKCC. The remainder of the patients had the tests sent to their homes and they returned the tests to our facility. The patients’ charts were also reviewed at the time of testing for birth and surgical procedure dates. We employed several robust and time consuming assessment tools to aid in measurement of psychological outcomes and quality of life. The Quality of Life Questionnaire (QLQ) is a 192 item, group form self-report measure, which takes approximately 30–45 min to complete was purchased from Multi-Health Systems (Toronto, ON, USA). The test items are used in the scoring of 15 content scales and a social desirability sale. The content scales are used to comprise a single overall score representing the patient’s assessment of quality of life. The domains assessed include material well-being, physical well-being, personal growth, marital relations, parent-child relations, extended family relations, extramarital relations, altruistic behavior, political behavior, job satisfiers, job characteristics, creative-esthetic behavior, and sports activity (29). Each question consists of a statement, which is answered either true or false. Intercorrelations with two independent inventories of physical health and general well-being were all statistically significant (29). The QLQ is validated for patients aged 18 and above (29). The MMPI is a psychological personality measurement device that assesses an array of psychological states and traits such as dysphoria, anxiety, ego strength, substance abuse proclivity, somatic concerns, and socialization (National Computer Systems, University of Minnesota, Minneapolis, MN, USA) (30, 31). Detailed descriptions of the different domains can be found in the manuals and handbook accompanying the assessment (30, 31). The questionnaire requires approximately 90–120 min to complete. The MMPI adolescent version is validated for patients 14–18 years of age and the adult version is validated for those over the age of 18 (30, 31). The validity scales include measures designed to determine if the subject is attempting portray themselves in a negative manner (K-Defensive) or alternatively filling out the questionnaires carelessly (VRIN-variable response inconsistency and TRIN-true response inconsistency). The questionnaire includes measures of anxiety, depression, obsessiveness, health concerns, anger, cynicism, self-esteem, aspirations, conduct, social discomfort, and negative treatment indicators (30, 31). The demographic assessment was a written questionnaire which gathered information about the patients’ education level, employment status, occupation, marital status, and annual income, where patients were able to fill in blanks. A Visual Analog Scale was used as a pain assessment where patients answered three questions about the extent of the pain experienced by placing a mark on a 100-mm horizontal line. The 0 mm end was designated the minimum and the 100-mm end labeled as the maximum pain or interference experienced. The questions asked include the following: amount of pain felt on average over the past month, amount of pain that interferes with daily activities over the past month, and amount of pain that interferes with sleep. The Visual Analog Scale has been validated for patients greater than 5 years old (32).

Statistical analysis was done using STATA 9.2 (StataCorp, College Station, TX, USA). The primary analysis, the difference of the main outcome variable, Total Quality of Life (part of the QLQ), was analyzed between the two groups. For each group comparison, the statistical test used was a two-sample Wilcoxon Rank Sum (Mann Whitney), chi-square test, a two-sample *t* test with equal or unequal variances, or a two-sample test of proportion. There were no confounding factors or effect modification observed. The study was designed to detect at least a 10% difference in the total quality of life yielding a power of 92%. Using a simple linear regression model between the main outcome and the other measured outcome categories, the factors that affected the Total Quality of Life were examined.

## Results

Eighty-three patients agreed to enroll on the study of a planned total of 100, but one patient did not complete all the assessments. The characteristics of the 82 patients who participated in the study are summarized in Table 1. The majority had been treated with limb-preservation procedures (*n* = 48) and the remainder having had amputations (*n* = 34). Demographic and medical information were obtained and quality of life and psychological outcomes were scored. All of the patients completed the questionnaires described in the Section “Patients and Methods.” There were no statistically significant differences in age range, sex, or time since surgery between the two groups used in the study. The percentage of males among the amputee group was 52.9% and 52.1% in limb-preservation patients. The limb-preservation patients demonstrated a greater education level, which was statistically significant with a *p*-value of 0.03. No other measures were statistically significant. The mean value for pain as indicated on a 10-cm scale was 2.11 for limb-preservation patients and 2.45 for amputees. Although there were slight differences in activity and sleep interference and pain levels between both groups, none were statistically significant.

**Table 1:** **Characteristics of patients with lower extremity bone sarcoma**.

	Amputation	Limb preservation	*P*-value
No. of subjects	34	48	
Age (mean ± SD, range)	27.6 ± 10.4 (14.9–49.9)	25.6 ± 8.3 (14.0–49.5)	0.51
Sex (M)	18 (52.9%)	25 (52.1%)	0.94
Age at surgery (mean, SD, range)	19.5 ± 9.7 (7.0–46.7)	20.0 ± 7.7 (9.8–44.6)	0.30
Years since surgery (mean, SD, range)	8.1 ± 6.6 (1.1–23.4)	5.6 ± 4.0 (1.1–16.9)	0.20
Employment
Employed	16 (47.1%)	31 (64.6%)	0.11
Marital status
Married	5 (14.7%)	14 (29.2%)	0.58
Education
Less than high school	8	11	0.03
Graduated high school	6	9	
Higher than high school	20	27	
Pain (mean, SD, range)	2.5 ± 2.6 (0–10)	2.1 ± 2.5 (0–8)	0.39
Activity interference (mean, SD, range)	2.1 ± 2.5 (0–10)	2.2 ± 2.6 (0–9)	0.92
Sleep interference (mean, SD, range)	1.7 ± 2.6 (0–10)	1.2 ± 2.0 (0–7)	0.45

The QLQ revealed significant differences in material well-being, occupational relations, creative-esthetic behavior, and sports activity (Table 2). Social desirability revealed a trend favoring limb-preservation surgery (*p*-value = 0.06).

**Table 2:** **Comparison of the quality of life questionnaire of patients with amputation and limb preservation**.

Category	Amputees (mean ± SD, range) *N* = 34	Limb preservation (mean ± SD, range) *N* = 48	*P*-value
Overall QOL	100.7 ± 27.5 (37–151)	115.9 ± 24.2 (63–168)	<0.01
Material well-being	36.7 ± 12.3 (6–52)	44.2 ± 12.6 (16–62)	<0.01
Occupational relations	45.9 ± 10.1 (22–64)	52.3 ± 11.9 (22–64)	<0.01
Job satisfiers	46.6 ± 8.2 (32–64)	54.19 ± 7.49 (38–68)	<0.01
Creative-esthetic behavior	50.3 ± 10.4 (30–68)	54.83 ± 8.95 (38–72)	0.04
Sports activity	46.1 ± 9.5 (34–68)	50.9 ± 10.6 (34–68)	0.04
Social desirability	42.7 ± 10.3 (26–64)	47.5 ± 11.8 (22–68)	0.06
Vacation behavior	43.8 ± 13.2 (18–64)	49.2 ± 10.3 (32–64)	0.12
Physical well-being	43.3 ± 11.9 (20–62)	46.8 ± 12.5 (10–68)	0.13
Extended family relations	49.5 ± 9.9 (30–64)	52.3 ± 10.2 (26–64)	0.15
Marital relations	47.1 ± 15.1 (18–62)	52.4 ± 9.0 (30–62)	0.16
Extramarital relations	51.2 ± 10.9 (18–66)	54.4 ± 8.7 (30–66)	0.17
Job characteristics	46.9 ± 7.9 (34–60)	49.6 ± 8.3 (30–60)	0.23
Altruistic behavior	44.9 ± 10.3 (30–68)	47.5 ± 9.2 (30–68)	0.24
Personal growth	47.9 ± 10.9 (18–64)	50.1 ± 10.2 (26–64)	0.36
Political behavior	46.2 ± 9.0 (32–64)	47.1 ± 11.3 (30–68)	0.76
Parent-child relations	52.4 ± 8.5 (42–62)	51.8 ± 9.3 (34–62)	0.88

*The overall quality of life is a *T*-score derived from all the sub-groups*.

*Average T-scores are 45–50*.

The MMPI detected relatively few differences between the groups (Table 3). The only domains in the Adult MMPI that depicted notable differences were (K) defensiveness, where the limb-preservation mean score was significantly higher, and (Mt) college maladjustment, where the amputees mean score was significantly lower. There were no statistically significant differences noted between the groups on the adolescent MMPI (results not shown) likely due to the small number of adolescents with amputation in the study.

**Table 3:** **Comparison of the adult MMPI of patients with amputation and limb preservation**.

Category	Amputees (mean ± SD, range) *N* = 30	Limb preservation (mean ± SD, range) *N* = 38	*P*-value
K (defensiveness)	53.6 ± 8.7 (35–68)	59.5 ± 10.2 (38–83)	0.01
Mt (college maladjustment)	52.3 ± 11.8 (33–84)	45.3 ± 11.7 (16–78)	0.01
D (depression)	61.3 ± 15.1 (36–104)	57.8 ± 13.4 (39–92)	0.31
Sc (schizophrenia)	64.1 ± 14.0 (42–111)	60.4 ± 11.1 (40–95)	0.33
Mf-f (masculinity–femininity) for females	52.0 ± 11.0 (34–74)	48.5 ± 11.4 (32–66)	0.38
F (infrequency)	58.8 ± 14.4 (44–102)	54.6 ± 9.1 (44–82)	0.40
Ma (hypomania)	59.6 ± 10.7 (40–83)	57.6 ± 14.2 (25–91)	0.53
Mf-m (masculinity–femininity) for males	61.2 ± 7.15 (49–73)	62.5 ± 8.1 (45–74)	0.63
Pd (psychopathic deviate)	59.9 ± 12.3 (39–90)	58.7 ± 10.9 (41–90)	0.67
Hy (conversion hysteria)	59.8 ± 10.8 (44–86)	60.8 ± 7.3 (47–76)	0.68
Hs (hypochondriasis)	60.0 ± 13.8 (39–95)	59.0 ± 9.1 (44–82)	0.70
Si (social introversion)	51.1 ± 10.0 (36–73)	50.3 ± 9.0 (30–68)	0.72
Pt (psychasthenia)	58.5 ± 11.3 (42–95)	58.4 ± 10.8 (41–85)	0.91
Pa (paranoia)	60.5 ± 12.6 (38–88)	60.5 ± 7.1 (50–85)	0.96

*T-scores and their correlation with each category: 85–90 extremely high, 75–80 very high, 65–70 high, 55–60 moderately high, 45–50 average, 35–40 moderately low, 30 very low*.

## Discussion

The purpose of this study was to compare the quality of life of survivors who underwent either amputations or limb-preservation procedures for lower extremity bone sarcoma. Our hypothesis was that sparing a person’s limb, as opposed to amputating it, would result in a better quality of life. Our main outcome variable, Total Quality of Life in the QLQ, demonstrated a statistically significant superior quality of life for the patients with the limb-preservation surgery. The QLQ demonstrated that the limb-preservation patients showed marked superiority to amputee patients in the areas of material well-being, occupational relations, job satisfiers, and sports activity; the majority of the other categories measured showed no statistical differences. The Minnesota Multiphasic Personality Inventories failed to detect any major difference, aside from a higher defensiveness score noted in the adult limb-preservation patients and a lower maladjustment score in the adult limb-preservation patients. Of a total potential planned accrual of 100 patients (50 limb-preservation patients and 50 amputee patients); 82 patients were accrued to participate in the study. The accrual was adequate to detect a statistical significance in the total quality of life domain. Our study demonstrates that there is a benefit to limb-preservation surgery as compared to amputation in regards to several psychological factors and total quality of life.

In 1982, Sugarbaker et al. analyzed 26 patients with soft tissue sarcoma who received either limb-preservation surgery or amputation utilizing several tests: Psychosocial Adjustment to Illness Scale, the Sickness Impact Profile, the Barthel Function Scale, and the Katz activities of Daily Living Scale (14). Although no differences were found, the sample size was very small resulting in a study with limited power which carries a high risk of type II error (14). In 1985, Weddington et al. compared amputees and patients with preserved limbs in terms of cognitive functioning, affect, mood, body image, physical functioning, global psychological adjustment to illness and surgery, and lifetime prevalence of psychiatric disorders (15). No significant differences were recorded between the two groups in this study (15). In 1992 Postma et al. studied long-term survivors of extremity sarcoma and compared patients who had received limb-preservation surgery to those who had undergone amputations (16). Psychoneurotic and somatic distress, activities of daily living, self-esteem, and adjustment to illness were compared in the two groups. Although differences were detected, they were not found to be significant (16). Chang et al. investigated patients treated for extremity sarcoma with limb-sparing surgery longitudinally for changes in psychosocial functioning and quality of life (17). A questionnaire designed by the investigators which included an economic assessment, an assessment of sexual functioning, the McGill Pain Questionnaire, and the Functional Living Index-Cancer were administered to patients at 6 month intervals with the maximum follow up being 42 months. Over time, significant changes were noted in almost all domains tested including employment status, sexual functioning, pain, and global quality of life (17). Pardasaney et al. showed an advantage of limb-preservation over amputation related to functional quality of life (limping, unable to drive, etc.) (34). Ginsberg et al. revealed that there were consistent differences in functional mobility assessment among patients who had either amputation, limb-preservation, and rotationplasty (35). Tabone et al. observed lower results for girls, physical functioning, and self-esteem if they had an endoprosthesis, and for family activity and pain associated with relapse (18). More recently, Zabrack et al. noted that patients with limb amputation had a significantly lower distress and anxiety when compared to all other solid tumor survivors (36). Robert et al. noted that patients’ quality of life was related to the functional aspect of the limb regardless of the type of surgery (37). Barrera et al. showed that patients with limb-preservation surgery struggle with sexual function as well as poor self-perception and depressive symptom (38). In a subsequent study, Barrera et al. noted that limb-preservation surgery, female gender, and older age were risk factors for reduced health related quality of life (39).

Quality of life in patients with extremity sarcoma is affected by limb preservation and amputation procedures. The presumed differences in psychosocial outcome and overall quality of life suggest that there is an advantage of quality of life of limb-preservation compared to amputation that was statistically significant in this study, in contrast to prior studies. The significance of this study is that it shows that there may be a direct benefit to limb-preservation surgery as compared to amputation in regards to total quality of life and identifies a few psychological factors that could be affected by surgery, mainly defensiveness.

For the QLQ, the limb-preservation patients had a higher mean value in every domain except parent-child relations. The statistically significant differences were interesting in that the limb-preservation patients had higher job satisfaction and occupational relations, suggesting that they are happier in their jobs. It may not be surprising that the sports activity is lower for amputation patients (33). Although not quite reaching statistical significance, social desirability is a domain worth analyzing. The way that patients view themselves in society is critical to the importance of limb-preservation surgery in regards to being cosmetically pleasing to the person. Even though limb-preservation patients had a higher score, it was still lower than the average person that confirms the finding that patients with limb-preservation surgery have issues with poor self-perception. Marital and employment status are statistically significant areas in reference to the main outcome variable, total quality of life. Limb-preservation patients are more likely to be married and employed than the amputation patients.

Possibly the most significant finding in this study is the higher defensiveness score for patients with limb preservation. For the MMPI, the limb-preservation patients scored significantly higher than the patients that had amputations in the *K* scale, also referred to as the defensiveness scale. A higher score suggests that patients with limb-preservation revealed less on the questionnaire, compared to patients who underwent limb amputation. Cognitive dissonance could explain the reason that the amputation group was not as defensive. Cognitive dissonance theory suggests that people feel distress when there is inconsistency in our beliefs and actions. One will then try to resolve this distress by changing his or her beliefs, actions or even just the perception of the actions. The amputee group may feel that removal of the affected limb has eliminated the chance of recurrence, and therefore may have greater peace of mind and less psychological distress compared to the limb-preservation patients. Such dissonance may also explain why prior studies suggest those with amputation have less anxiety, especially after adjustment for demographic variables (36).

One limitation of our study is the small number of adolescent patients, especially patients who received amputation (*n* = 4). Although limb-preservation surgery has come to dominate the surgical approach to extremity sarcoma, extent of disease, neurovascular involvement, surgical preference, and the patient’s preconceived preference for surgery or psychological profile may have influenced the choice of the procedure. The physicians and surgeons are instrumental in molding patient expectations and this could alter long-term psychological outcome. By recognizing this potential bias, a psychologist could intervene to help patients better understand their own decisions of amputation versus limb-preservation and their long-term expectations. There may have been selection bias in which subsequent quality of life differences were secondary to baseline characteristics. These baseline features include factors such as the size of the tumor and the extent of disease spread that might influence the outcome in ways that cannot easily be identified. It will not be possible to eliminate this limitation in the context of this study. The MMPI measures personal, social, and behavioral issues. Life-altering surgery could easily affect these areas. We cannot exclude a pre-existing psychological condition prior to surgery. The clinical scales of the MMPI variables present some difficulty in the analysis. We can speculate that the psychological profile of an individual should remain stable over time whereas the quality of life of patients differs more over time. This could explain our failure to detect any statistically significant difference in the MMPI. Another limitation to our study is that we are unsure how non-response bias has influenced the results. Although the adolescent group was a small number, we included their results in the measures that were not validated for their age group since excluding them in the final analysis did not produce any statistically significant changes in the results. A final limitation of our study is multiple comparisons that may lead to false positive results. Our study must be viewed as exploratory to identify domains that differ between the groups that can be validated in a subsequent study. We detected a significant difference in Overall Quality of Life between the two groups, without necessitating subgroup or sub-domain analyses.

There are multiple quality of life measures and the prior studies have been inconsistent in their results. In choosing a much longer and more robust measure for the quality of life, we have erred on the side of sensitivity to detect a difference in our patient population and therefore needs to be further validated in a future study. One main challenge that we hoped to accomplish was measuring the psychological outcomes of young adult cancer survivors utilizing the MMPI-A. However, our study did not have enough adolescent patients to have any statistically significant outcomes.

Based on this study and coupled with the results of the prior studies (34, 35, 37), lower extremity limb-preservation patients have better functional outcomes and quality of life than amputation patients. Future quality of life orthopedic outcome procedure comparison studies of bone tumor survivors will be difficult, as the majority of patients at present have limb-preservation surgery making the accrual of patients with amputation time consuming and potentially biased. The accrual of these rare patients would mandate a multi-institutional study. Further studies should be geared toward confirming the statistically significant values that affected overall quality of life as identified in this study with the concept of cognitive dissonance underscoring the results.

## Conflict of Interest Statement

The authors declare that the research was conducted in the absence of any commercial or financial relationships that could be construed as a potential conflict of interest.
